# Policy Series: Dementia Care Policy and Practice: Early Detection, Living Alone, and Nonpharmacological Interventions

**DOI:** 10.1093/geroni/igaf122.1723

**Published:** 2025-12-31

**Authors:** Michael Lepore, Tara McMullen, Ian Kremer

## Abstract

Dementia is a public health priority with a wide range of implications for policy and practice. Nations, states, and local communities address dementia in many different fields of policy and practice. This symposium builds on a recent thematic issue of Public Policy & Aging Report to address important opportunities and challenges for policy and practice regarding dementia, including arguments for and against early detection, gaps in care for people with dementia who live alone, and measuring outcomes of nonpharmacological interventions. The symposium comprehensively reviews the policy landscape for dementia and by focusing on unique challenges and opportunities related to dementia care, this symposium brings to light more and less successful policy and practice initiatives, highlights emerging issues and concerns, and provides a forum for discussion and policy debate, including discussion of alternative policy options. This symposium will serve as a launchpad for disseminating new knowledge on dementia care policy and practice, facilitating dementia care policy and practice comparisons and collaboration, and shaping the future of policy and practice regarding care for people living with dementia.

